# Echocardiographic assessment of left ventricular longitudinal function in critically ill patients

**DOI:** 10.1186/s13054-026-06193-5

**Published:** 2026-07-10

**Authors:** Oscar Cavefors, Odd Bech-Hanssen, Björn Redfors, Jonatan Oras

**Affiliations:** 1 Department of Anaesthesiology and Intensive Care Medicine, Institute of Clinical Sciences, Sahlgrenska Academy, University of Gothenburg, Gothenburg, Sweden; 2 Department of Anaesthesiology and Intensive Care Medicine, Sahlgrenska University Hospital, Gothenburg, Sweden; 3 Department of Molecular and Clinical Medicine, Institute of Medicine, Sahlgrenska Academy, University of Gothenburg, Gothenburg, Sweden; 4 Department of Clinical Physiology, Sahlgrenska University Hospital, Gothenburg, Sweden; 5 Department of Cardiology, Sahlgrenska University Hospital, Gothenburg, Sweden

## Abstract

**Background:**

In the intensive care unit (ICU), conventional assessment of left ventricular systolic function relies on left ventricular ejection fraction (LVEF), but LVEF often has limited prognostic ability. Measures of longitudinal LV performance [global longitudinal strain (GLS), mitral annular plane systolic excursion (MAPSE), and tissue Doppler–derived systolic velocity (S’)] are increasingly used in critical care echocardiography and may capture clinically relevant dysfunction better than LVEF. To date, the prognostic implications of GLS, MAPSE, and S’ have been studied mainly in septic cohorts, while their feasibility and prognostic value in mixed ICU populations remain uncertain. We therefore aimed to evaluate the feasibility and prognostic value of these parameters in a mixed ICU cohort.

**Methods:**

In this exploratory secondary analysis of a prospective observational ICU cohort, transthoracic echocardiography was performed within 24 hours of ICU admission. LVEF by Simpson biplane, GLS, MAPSE, and S’ were analysed offline. Feasibility was quantified as the proportion of patients with analysable measurements. Associations between echocardiographic parameters were assessed using simple linear regression and are reported as coefficients of determination (R^2^). Associations with 90-day mortality were assessed using logistic regression, with adjustment for age, Simplified Acute Physiology Score 3, and cardiac index.

**Results:**

Of 411 enrolled patients, 377 had at least one parameter available and were included. Feasibility was 71% for LVEF, 65% for GLS, 90% for MAPSE, and 83% for S’. GLS correlated most strongly with LVEF (R^2^=0.516), whereas correlations were weaker for MAPSE (R^2^=0.268) and S’ (R^2^=0.195). Ninety-day mortality was 27% (101/377). After adjustment, GLS (odds ratio [OR] 1.08 per 1% less negative strain, 95% confidence interval [CI] 1.00–1.16; p=0.048) and MAPSE (OR 1.17 per 1 mm decrease, 95% CI 1.06–1.30; p=0.002) remained associated with mortality, whereas LVEF and S’ did not. MAPSE ≤10 mm was independently associated with mortality (adjusted OR 2.48, 95% CI 1.31–4.71; p=0.005).

**Conclusions:**

In a mixed ICU cohort, impaired MAPSE and GLS were associated with increased mortality, in contrast to LVEF and S’. MAPSE was also the most feasible measurement. Incorporation of MAPSE into routine ICU echocardiography may improve detection and risk stratification of LV dysfunction in critical illness.

**Trial registration:**

Secondary analysis of data from a single-centre prospective observational study focused on systolic dysfunction in ICU patients (Clinical Trials ID: NCT03787810)

**Supplementary Information:**

The online version contains supplementary material available at https://doi.org/10.1186/s13054-026-06193-5.

## Introduction

Left ventricular ejection fraction (LVEF) remains the most widely used echocardiographic parameter to evaluate left ventricular systolic function. However, its clinical applicability in critically ill patients is limited. Quantitative assessment of LVEF is technically demanding, shows considerable intra- and interobserver variability, and is strongly dependent on loading conditions that frequently fluctuate in the intensive care setting [1–3]. These limitations underscore the need for alternative measures of systolic function. Parameters reflecting longitudinal myocardial motion, such as global longitudinal strain (GLS), mitral annular plane systolic excursion (MAPSE), and tissue Doppler-derived systolic velocity (S’), may offer more robust and feasible assessments [4–8]. The Preferred Reporting Items for Critical Care Echocardiography Studies (PRICES) consensus statement recommends reporting these parameters in studies of critically ill patients [9].

Several studies in patients with sepsis and COVID-19 have demonstrated that impaired GLS is consistently associated with increased mortality and a recent meta-analysis confirmed GLS as a prognostic marker in sepsis [4, 7, 10, 11]. MAPSE and S’ have also been linked to adverse outcomes in smaller studies and are simpler to acquire than GLS, but evidence is sparse and largely confined to sepsis [5, 10, 12–14]. Thus, while GLS, MAPSE, and S’ appear promising, their prognostic value in heterogeneous ICU populations remains unclear.

The aim of this study was to evaluate the clinical utility of GLS, MAPSE, and S’ in a heterogeneous cohort of critically ill patients. Specifically, we sought to assess their feasibility, relationship with conventional measures of systolic function (LVEF), and prognostic value for 90-day mortality. We also examined their performance across clinically important subgroups (sepsis, respiratory failure, cardiac disease).

## Methods

### Design

The study is an exploratory secondary analysis of previously gathered intensive care cohort data for a prospective observational trial on left ventricular (LV) systolic function (Clinical Trials ID: NCT03787810), approved by the Regional Ethics Committee in Gothenburg, Sweden (registration number 036-18) [15]. In the original study, 411 patients were included between May 28, 2018, and January 20, 2019. A secondary analysis from the same dataset reported on left ventricular diastolic function [16].

### Patients

#### Patient population

Patients were prospectively included from the general and neuro ICU at Sahlgrenska University hospital, a tertiary university hospital. The general ICU admits critically ill patients from all medical and surgical specialties, except for postoperative cardiothoracic patients and those receiving mechanical circulatory support. The neuro ICU primarily treats patients with neurosurgical or neurological conditions. Both units manage a mix of unselected local admissions and tertiary referrals.

#### Inclusion criteria

The inclusion criteria for the original study were: age > 18 years, transthoracic echocardiography (TTE) within 24 hours of ICU admission and a Sequential Organ Failure Assessment (SOFA) score greater than one [17]. For this secondary analysis, only patients with echocardiographic images of adequate quality for post-processing were included.

For the purposes of this study, patients were categorized into four diagnostic groups: sepsis, respiratory failure, cardiac disease, and other. The following definitions were applied:

- Sepsis: Admission due to sepsis, as defined by current clinical guidelines [18].
- Respiratory failure: Defined pragmatically as hypoxemic respiratory failure with PaO₂/FiO₂ <40 kPa. This threshold was used to identify clinically relevant impaired oxygenation and was not intended to define ARDS.
- Cardiac disease: History of heart failure, coronary artery disease, significant valvular heart disease, or acute cardiac events such as myocardial infarction.
- Other: Not fulfilling any of the criteria above, i.e., ICU admission without sepsis, respiratory failure or cardiac disease.

### Echocardiographic examination and analysis

A TTE, according to guidelines, was performed by an experienced operator within 24 hours of ICU admission [19, 20]. Examinations were conducted after initial cardiovascular stabilization and volume resuscitation. The majority of the examinations were performed using either a Vivid S70 ultrasound system with an M5Sc-D matrix array transducer or a Logiq E9 system (GE Healthcare, Milwaukee, WI, USA).

Data on LVEF, regional wall motion abnormalities, and cardiac output (CO) measurements were collected as part of the original study. For the present study, previously acquired echocardiographic images were reanalysed offline using EchoPac software (GE Healthcare, Milwaukee, WI, USA). GLS was assessed using speckle-tracking echocardiography in three standard apical views: apical three-chamber (3CH), four-chamber (4CH), and two-chamber (2CH). A 17-segment model was employed for strain analysis. Images were considered invalid if more than two segments in any single view were deemed unsuitable for analysis [20]. A GLS cutoff value of –16% was used to define impaired systolic function [21].

MAPSE was measured using M-mode at septal and lateral annular positions in the 4CH view. Tissue Doppler imaging (TDI)-derived mitral annular systolic velocity (S’) was measured using pulsed-wave Doppler at the septal and lateral annulus in the apical 4CH view. Patients with regional wall motion abnormalities were included. Measurements of MAPSE and S’ were averaged over three cardiac cycles in patients with regular rhythms and over five cycles in those with irregular rhythms. If both septal and lateral values were available, an average was used.

### Clinical data

At the time of the TTE, relevant clinical variables were recorded, including ventilator settings, arterial blood pressure, heart rate, vasopressor and inotropic dosages, serum creatinine, lactate levels, and the PaO₂/FiO₂ ratio. Additional demographic and clinical data were extracted from the medical records, including age, sex, medical history, SOFA score and Simplified Acute Physiology Score 3 (SAPS 3) [17, 22]. The occurrence of cardiac arrest, myocardial infarction, and sepsis (both suspected and confirmed) were documented according to established clinical guidelines [23]. As outcome measures, time to death during the first 90 days and mortality at day 90 was recorded.

### Statistics

Continuous variables are presented as mean ± standard deviation (SD) or median with interquartile range (IQR), and categorical variables as counts and percentages. Group comparisons were performed for each clinical condition (sepsis, respiratory failure, and cardiac disease) versus patients without the respective condition (group Other). Continuous variables were compared between two groups using the t-test or Mann–Whitney U test, while categorical variables were compared using Fisher’s exact test. Associations between echocardiographic parameters were assessed using simple linear regression and are reported as coefficients of determination (R^2^). Logistic regression was used to assess associations between echocardiographic parameters and 90-day mortality, adjusting for age, SAPS 3 score, and cardiac index [22, 23]. To assess potential bias related to missing data, clinical characteristics were compared between patients with and without feasible GLS, MAPSE, and S′ measurements using the statistical methods described above. The incremental prognostic value of GLS and MAPSE was assessed using nested logistic regression models and likelihood ratio tests based on the −2 log-likelihood (−2LL). The baseline model included age, SAPS 3 score, and cardiac index, with GLS and MAPSE added individually and jointly. Statistical analyses were performed using IBM SPSS Statistics (IBM Corp., Armonk, NY, USA), with p < 0.05 considered significant. No formal sample size calculation was performed.

## Results

**Fig. 1:**
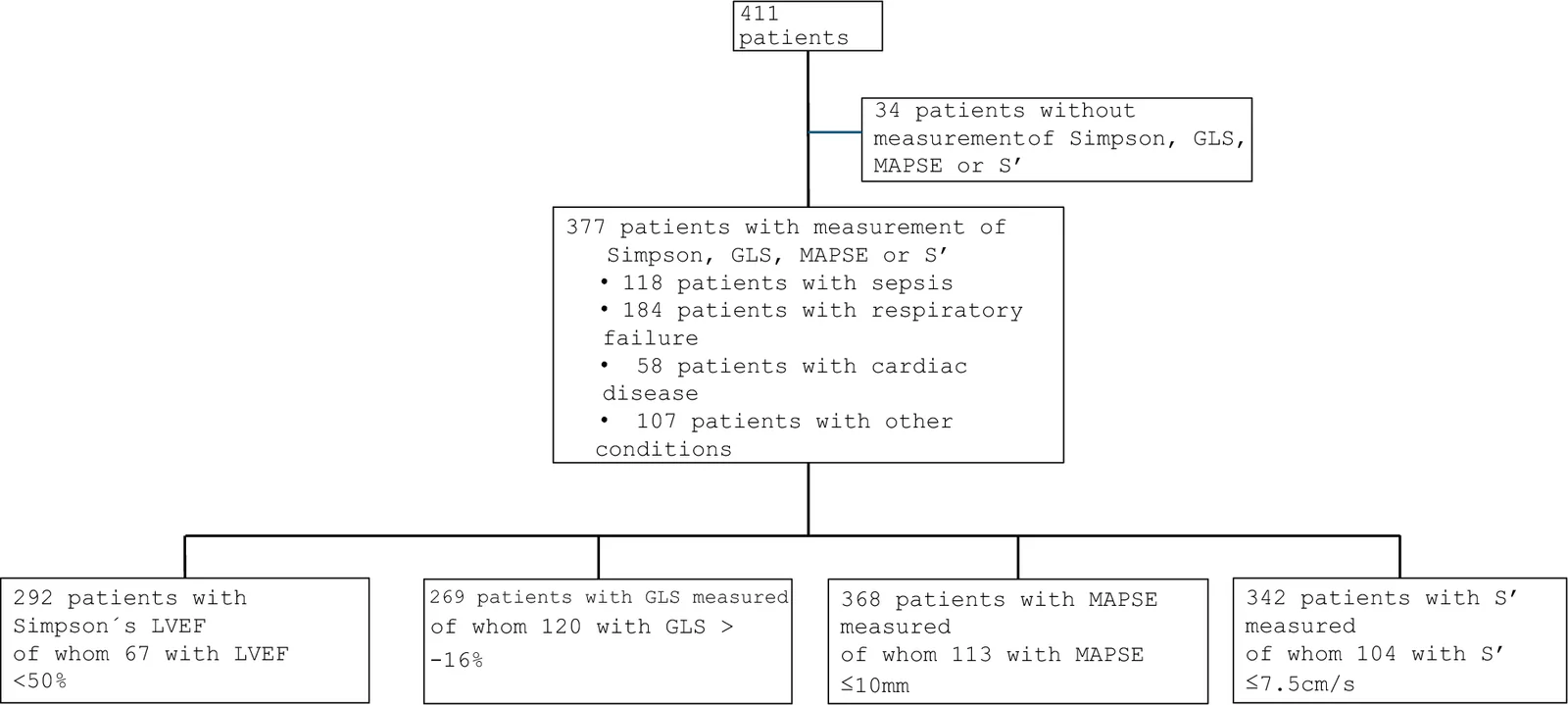
Flow chart of patient inclusion and availability of echocardiographic measurements. GLS, global longitudinal strain; MAPSE, mitral annular plane systolic excursion; S’, mitral annular systolic velocity

A total of 411 patients were enrolled between May 2018 and January 2019. Of these, 377 had at least one measurement of LVEF using Simpson biplane, GLS, MAPSE, or S’ available and were included in the present analysis (Fig. 1). In a missing data analysis, patients with and without feasible GLS, MAPSE, and S′ measurements did not differ significantly with respect to SAPS 3 score, cardiac index, age, BMI, or mechanical ventilation.

The median age of the whole cohort was 65 years, and 60 % were male. The median SAPS 3 score at admission was 58. Patients with sepsis were older and had the highest illness severity compared with the Other group. Patients with respiratory failure also had higher SAPS 3 scores and a higher prevalence of pulmonary disease and diabetes. The cardiac disease group was the oldest subgroup and had the highest prevalence of coronary artery disease and arrhythmias. Patients categorized as Other were younger and most often admitted due to neurological conditions (Table 1).

**Table 1:** Baseline characteristics and reasons for ICU admission according to clinical subgroup

Category	Variable	Sepsis n=118	Respiratory failure n=184	Cardiac disease n=58	Other conditions N=107
Demographics	Male sex, n (%)	62 (53)	113 (61)	41 (71)	67 (63)
	Age, years	70 (57 - 76)	67 (55 - 74)	73 (69 - 79)	60 (39 - 70)
	BMI	26 (23 - 30)	27 (24 - 31)	26 (23 - 28)	25 (23 - 27)
Risk scores	SAPS3 score	67 ± 14	62 ± 16	60 ± 13	51 ± 15
Medical history	Hypertension, n (%)	46 (39)	76 (41)	33 (57)	23 (21)
	Diabetes, n (%)	19 (16)	28 (15)	14 (24)	21 (20)
	Hyperlipidaemia, n (%)	13 (11)	19 (10)	11 (19)	5 (5)
	Heart failure, n (%)	9 (8)	11 (6)	17 (29)	0 (0)
	Myocardial infarction, n (%)	8 (7)	12 (7)	23 (40)	0 (0)
	Coronary artery disease, n (%)	14 (12)	23 (13)	39 (67)	0 (0)
	Any cardiac disease, n (%)	20 (17)	34 (18)	58 (100)	0 (0)
	Arrhythmias, n (%)	16 (14)	18 (10)	16 (28)	6 (6)
	Valve disease, n (%)	4 (3)	4 (2)	4 (7)	0 (0)
	COPD, n (%)	12 (10)	19 (10)	11 (19)	1 (1)
	Pulmonary disease, other than COPD, n (%)	15 (13)	32 (17)	14 (24)	4 (4)
	Renal disease, n (%)	9 (8)	14 (8)	8 (14)	3 (3)
	Liver disease, n (%)	8 (7)	15 (8)	2 (3)	18 (17)
	Cerebrovascular disease, n (%)	12 (10)	16 (9)	8 (14)	6 (6)
	Neurological disease, n (%)	11 (9)	17 (9)	4 (7)	10 (9)
	Endocrinal disease, n (%)	6 (5)	11 (6)	3 (5)	5 (5)
	Rheumatologic disease, n (%)	12 (10)	20 (11)	3 (5)	6 (6)
	Alcohol overconsumption, n (%)	12 (10)	14 (8)	5 (9)	7 (7)
	Psychiatric disease, n (%)	13 (11)	20 (11)	3 (5)	12 (11)
	Malignancy, n (%)	17 (14)	22 (12)	6 (10)	9 (8)
Reason(s) for	Cardiovascular reason, n (%)	73 (62)	91 (50)	36 (62)	29 (27)
admission	Liver failure, n (%)	13 (11)	20 (11)	3 (5)	18 (17)
	Gastrointestinal reason, n (%)	23 (20)	20 (11)	3 (5)	6 (6)
	Neurological reason, n (%)	25 (21)	51 (28)	15 (26)	43 (40)
	Renal failure, n (%)	26 (22)	30 (16)	7 (12)	9 (8)
	Respiratory reason, n (%)	70 (60)	88 (48)	23 (40)	15 (14)
	Haematological reason, n (%)	7 (6)	4 (2)	0 (0)	4 (4)
	Metabolic reason, n (%)	27 (23)	41 (22)	9 (16)	11 (10)
	Trauma, n (%)	3 (3)	12 (7)	6 (10)	16 (15)
	Other reason, n (%)	5 (4)	13 (7)	5 (9)	13 (12)
Sepsis	Sepsis, n (%)	118 (100)	79 (43)	20 (34)	0 (0)

BMI, Body mass index; SAPS 3, Simplified acute physiology score 3; COPD, Chronic obstructive pulmonary disease

Feasibility differed between the echocardiographic parameters. LVEF could be assessed by Simpson biplane method in 292 patients (71 %), GLS in 269 (65 %), MAPSE in 368 (90 %), and S’ in 342 (83 %). For MAPSE, 366 patients had septal, and 366 had lateral data available. For S’, 336 had septal, and 325 had lateral data available. Among those with analysable recordings, 67 patients (23 %) had LVEF < 50 %, 120 (45 %) had GLS > –16 %, 113 (31 %) had MAPSE ≤ 10 mm, and 104 (30 %) had S’ ≤ 7.5 cm/s (Fig. 1).

Patients with sepsis, respiratory failure, or cardiac disease had generally lower echocardiographic indices of left ventricular systolic function compared with patients without the respective condition. In sepsis, impaired GLS was the most frequent abnormality (58%), followed by reduced MAPSE (40%). Among patients with respiratory failure, GLS again showed the highest prevalence of dysfunction (55%). In the cardiac disease group, abnormalities were common across all parameters, with GLS (74%) and MAPSE (59%) being most affected. In contrast, systolic abnormalities were uncommon in the Other group (Table 2).

**Table 2:** Echocardiographic measures of left ventricular systolic function according to clinical subgroup

Category	Variable	Sepsis n=118	Respiratory failure n=184	Cardiac disease n=58	Other n=107
Continuous	Simpson biplane, %	57 ± 13*	55 ± 15*	49 ± 16*	64 ± 8
	GLS, %	15 ± 4,5*	15 ± 5.1*	12.3 ± 5.1*	19.8 ± 3.9
	MAPSE, mm	11.1 ± 3.1*	11.1 ± 3.4*	9.4 ± 3.3*	14.1 ± 2.7
	S’, cm/s	8.6 ± 2.9	8.5 ± 2.9*	7.4 ± 3.0*	9.8 ± 2.4
Binary	Simpson <50%, n (%)	19 (22)*	39 (29)*	24 (51)*	2 (2)
	GLS > -16%, n (%)	47 (58)*	75 (55)*	32 (74)*	12 (10)
	MAPSE ≤10mm, n (%)	46 (40)*	72 (40)*	35 (59)*	7 (7)
	S’ ≤7.5cm/s, n (%)	36 (33)	59 (36)*	24 (51)*	14 (14)

GLS, global longitudinal strain; MAPSE, mitral annular plane systolic excursion; S’, mitral annular systolic velocity. * Statistically significant compared to group “other”. Percentages are calculated from the number of patients with available measurements for each echocardiographic parameter

Using dichotomised cut-offs, the longitudinal systolic parameters (GLS, MAPSE and S’) generally discriminated patients with more severe clinical derangements. Patients with abnormal longitudinal function had, on average, higher heart rates, higher doses of noradrenaline, higher lactate levels, and lower P/F ratios. These parameters also showed clear separation across other echocardiographic indices associated with impaired cardiac dysfunction, including markers of elevated filling pressures (TR Vmax, e′, E/e′, LAVI) and reduced systolic performance (cardiac index, stroke volume index, LVOT VTI, TAPSE and fractional area change). Although the degree of discrimination varied between the individual longitudinal indices and across specific variables, the overall pattern consistently pointed toward worse physiologic and echocardiographic profiles in patients with impaired longitudinal function (Supplemental Table 1).

**Fig. 2:**
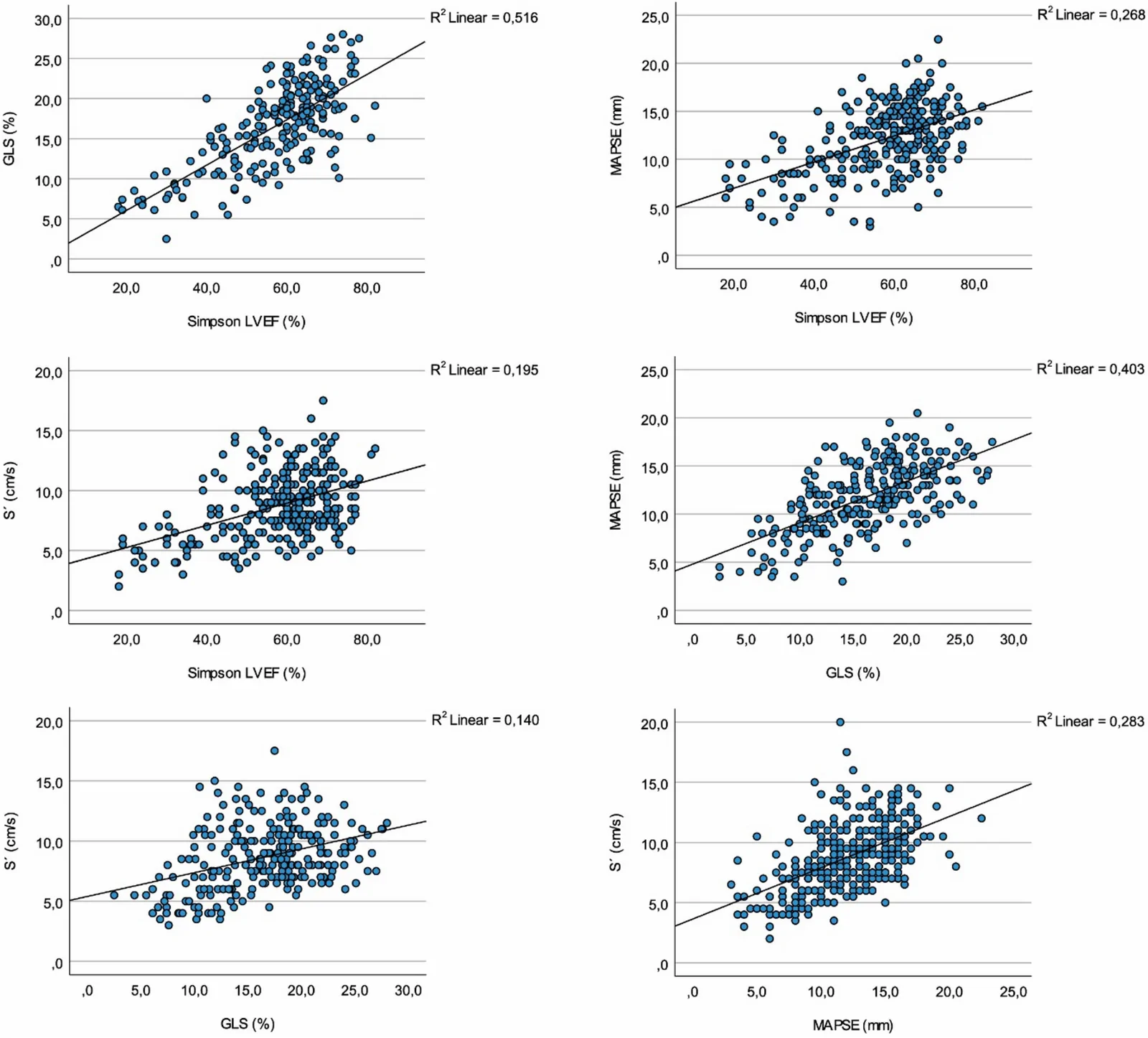
Correlations between the tested echocardiographic parameters. Simpson LVEF, Simpson biplane left ventricular ejection fraction; GLS, global longitudinal strain; MAPSE, mitral annular plane systolic excursion; S’, mitral annular systolic velocity

Correlations were observed between the echocardiographic measures of systolic function. GLS was most closely related to LVEF (R^2^ = 0.516), whereas the associations were weaker for MAPSE (R^2^ = 0.268) and S’ (R^2^ = 0.195). Moderate correlations were also noted between GLS and MAPSE (R^2^ = 0.403) and between MAPSE and S’ (R^2^ = 0.283) (Fig. 2).

At 90 days, 101 patients (27 %) had died. In unadjusted analyses, all echocardiographic parameters were associated with mortality. After adjustment for age, SAPS 3 score, and cardiac index, the associations remained significant for GLS (OR 1.08 per % increase, 95% CI 1.00–1.16, p = 0.048) and MAPSE (OR 1.17 per mm decrease, 95% CI 1.06–1.30, p = 0.002), but not for Simpson biplane LVEF (OR 1.11 per 10% decrease, 95% CI 0.87–1.42, p = 0.399) or S′ (OR 1.11 per cm/s decrease, 95% CI 0.98–1.25, p = 0.109). For dichotomized parameters, MAPSE ≤10 mm remained independently associated with mortality (adjusted OR 2.48, 95% CI 1.31–4.71, p = 0.005), whereas GLS > −16% (OR 1.62, 95% CI 0.76–3.49, p = 0.215), S′ ≤7.5 cm/s (OR 1.46, 95% CI 0.73–2.89, p = 0.282), and Simpson biplane LVEF <50% (OR 1.20, 95% CI 0.56–2.57, p = 0.648) were not significant (Table 3).

**Table 3:** Associations between echocardiographic parameters and 90-day mortality

Category	Echocardiographic parameter	Unadjusted			Adjusted*		
		OR	95% CI for OR	p-value	OR	95% CI for OR	p-value
Continuous	Simpson biplane, per 10% decrease	1.24	1.03 - 1.49	0.023	1.11	0.87 - 1.42	0.399
	GLS, per % increase	1.15	1.08 - 1.22	<0.001	1.08	1.00 - 1.16	0.048
	MAPSE, per mm decrease	1.26	1.17 - 1.37	<0.001	1.17	1.06 - 1.30	0.002
	S’, per cm/s decrease	1.16	1.06 - 1.27	0.002	1.11	0.98 - 1.25	0.109
Dichotomized	Simpson biplane <50%	1.89	1.05 - 3.37	0.033	1.20	0.56 - 2.57	0.648
	GLS > -16%	3.17	1.77 - 5.66	<0.001	1.62	0.76 - 3.49	0.215
	MAPSE ≤10mm	4.37	2.66 - 7.16	<0.001	2.48	1.31 - 4.71	0.005
	S’ ≤7.5cm/s	2.14	1.28 - 3.57	0.004	1.46	0.73 - 2.89	0.282

GLS, global longitudinal strain; MAPSE, mitral annular plane systolic excursion; S’, mitral annular systolic velocity; CI, confidence interval; OR, odds ratio

^*^ Adjusted for SAPS 3 score, age and cardiac index

In nested model analyses restricted to patients with complete datasets for GLS, MAPSE and cardiac index, MAPSE significantly improved the baseline model whereas GLS did not (Supplemental Analysis 1). However, when both variables were included simultaneously, neither GLS nor MAPSE provided significant incremental prognostic information beyond the other, suggesting substantial overlap between these measures.

Exploratory subgroup analyses were performed in patients with sepsis, respiratory failure, cardiac disease, and other conditions. Associations between impaired longitudinal systolic function and mortality were generally similar across subgroups, although confidence intervals were wide and statistical significance was not consistently retained after multivariable adjustment. The subgroup analyses are presented in Supplementary Table 2.

## Discussion

The main finding of this study is that echocardiographic parameters reflecting longitudinal systolic function, GLS and MAPSE, were associated with 90-day mortality in a mixed population of critically ill patients, whereas LVEF was not. Among these measures, MAPSE showed the strongest association with outcome and was also the most feasible parameter to obtain. These findings indicate that simple indices of longitudinal myocardial motion may provide prognostic information beyond conventional assessment of LVEF in the intensive care setting.

In this study, conventional assessment of systolic function by LVEF did not identify patients at increased risk of death, despite a substantial proportion showing mildly or moderately reduced values. Interestingly, LVEF was still associated with reduced cardiac output (demonstrated by lower cardiac index, SVI and VTI) and linked to classical signs of shock such as increased lactate and increased vasopressor requirement. These findings suggest that cardiac dysfunction, as demonstrated by reduced longitudinal markers, may be linked to increased mortality by other mechanisms than more pronounced circulatory instability.

Previous studies in septic patients have demonstrated similar findings, where GLS, but not LVEF, predicted outcome [4, 24]. Meta-analyses have confirmed GLS as a robust marker of mortality in sepsis [7, 25]. In line with this, our data extend these observations beyond sepsis, showing that the prognostic value of GLS may be retained in a mixed ICU population.

Despite its prognostic value, GLS has important limitations in critically ill patients. It requires adequate image quality in three apical views and is relatively time-consuming to acquire and analyse. In addition, GLS assessment assumes comparable loading conditions and sufficiently stable RR intervals across the different views, which may be difficult to achieve in ICU populations. GLS may also be influenced by cardiac and body size, and sex-specific differences have been reported in healthy populations [21, 26]. A larger healthy ventricle may therefore show lower absolute GLS values than a smaller ventricle, whereas this size dependency appears less pronounced for MAPSE [26].

In contrast to GLS, MAPSE is simple to acquire, highly feasible, and does not require advanced post-processing. In the present cohort, MAPSE correlated with GLS and remained independently associated with 90-day mortality, supporting its role as a pragmatic marker of longitudinal LV dysfunction in critically ill patients. Previous studies comparing MAPSE and GLS have yielded divergent findings across clinical settings and imaging modalities. In a large cardiac magnetic resonance cohort, Xue et al. reported that automated MAPSE showed stronger prognostic associations than feature-tracking GLS and LVEF [27]. In contrast, Gozdzik et al. found that strain-based measures, particularly exercise GLS, had greater prognostic utility than MAPSE and S′ in patients with HFpEF [28]. These apparently contrasting findings may reflect differences in patient populations, imaging modality, measurement technique, and clinical setting.

Previous critical care studies have also shown that MAPSE and related longitudinal displacement measures are associated with LV function and myocardial injury, and may contribute to outcome prediction [5, 12, 29–31]. However, conventional M-mode-derived MAPSE is limited by angle dependency, reliance on correct cursor alignment, and potential out-of-line annular motion [32, 33]. In addition, variability related to the sampled annular region should be considered [34, 35].

In the present ICU cohort, the clinical value of MAPSE lies in the combination of high feasibility and independent prognostic association. The overlap with GLS observed in the nested model analyses suggests that the two measures capture related aspects of longitudinal LV function, but not necessarily identical information. MAPSE may therefore be particularly useful as a pragmatic bedside marker when rapid and feasible assessment is needed.

For S’, emerging data support its prognostic relevance. In a large septic cohort, mitral S’ was only moderately correlated with LVEF, but independently associated with 28-day mortality [36]. It had a linear relationship to mortality that differs from the non-linear or “U-shaped” patterns sometimes reported for LVEF, suggesting that S’ may better reflect overall myocardial performance in severe illness [36, 37]. However, in our results S’ was not linked to an increased mortality after adjustments.

This study has several strengths. It represents one of the largest investigations of echocardiographic systolic parameters in a mixed population of critically ill patients, with standardized image acquisition and analysis performed within 24 hours of ICU admission. The simultaneous evaluation of GLS, MAPSE, and S’ allowed for direct comparison between established and more pragmatic measures of longitudinal function. Moreover, the inclusion of unselected ICU patients enhances the generalizability of the findings beyond specific diagnostic categories.

To reflect routine clinical practice, MAPSE and S′ were averaged over multiple cardiac cycles whereas GLS and LVEF were not. Although averaging GLS and LVEF over multiple cycles might have improved measurement precision, it would have reduced applicability to routine clinical practice.

Predefined dichotomous cut-offs were used to facilitate clinical interpretation, but dichotomization of continuous LV function parameters will result in loss of information and may obscure graded associations. These categorical analyses should therefore be interpreted as complementary to the continuous analyses. The subgroup analyses should also be interpreted cautiously due to limited statistical power. However, the generally consistent direction of associations across the different ICU subgroups suggests that the prognostic relevance of longitudinal systolic dysfunction may not be restricted to a specific diagnostic category.

Some limitations should be acknowledged. First, this was an exploratory secondary analysis and the analyses were not prespecified at study initiation. Second, missingness of echocardiographic measurements may not have been random. In critically ill patients, image quality and feasibility can be affected by factors such as mechanical ventilation, high airway pressures, lung overinflation, obesity, patient positioning, dressings, drains, and clinical instability. These factors may both reduce the likelihood of obtaining analysable echocardiographic images and be associated with disease severity and outcome. Although patients with and without feasible GLS, MAPSE, and S′ measurements did not differ significantly with respect to SAPS 3 score, cardiac index, age, BMI, or mechanical ventilation, selection bias due to non-random missingness and residual confounding cannot be excluded. Third, echocardiography was performed only once, within 24 hours of ICU admission. The measurements therefore represent a static assessment under prevailing treatment conditions and do not capture changes in cardiac function over time. As LVEF and longitudinal parameters are load- and context-dependent, ongoing vasopressor or inotropic therapy, fluid status, and mechanical ventilation may have influenced the results.

## Conclusion

In critically ill patients, measures of longitudinal systolic function provide prognostic information beyond conventional assessment of LVEF. Among these, MAPSE stands out as a highly feasible and clinically relevant parameter that can be obtained quickly and with minimal technical requirements. Incorporating this simple measurement into routine echocardiography may improve recognition of cardiac dysfunction in critical illness that could also be used for prognostication.

## Supplementary Information

Additional file 1.

## Data Availability

The datasets used or analyzed during the current study are available from the corresponding author upon reasonable request.
