# Supplementary material for: Echocardiographic assessment of left ventricular longitudinal function in critically ill patients
Source: Crit Care. 2026 Jul 10;30:365. doi: 10.1186/s13054-026-06193-5 (PMC13352900; doi:10.1186/s13054-026-06193-5)
Supplement: Supplementary file 1 — Additional file 1. [file 13054_2026_6193_MOESM1_ESM.docx]

|  | Simpson biplane | |  | GLS | |  | MAPSE | |  | S' | |
| --- | --- | --- | --- | --- | --- | --- | --- | --- | --- | --- | --- |
|  | EF ≥ 50%  (n=225) | EF < 50%  (n=67) |  | GLS ≤ -16%  (n=149) | GLS > -16%  (n=120) |  | MAPSE > 10mm  (n=255) | MAPSE ≤ 10  (n=113) |  | S' > 7.5cm/s  (n=238) | S' ≤ 7.5cm/s (n=104) |
| MAP, mmHg | 78 ± 13 | 78 ± 15 |  | 79 ± 13 | 77 ± 14 |  | 79 ± 14 | 76 ± 14 |  | 77 ± 13 | 81 ± 15 |
| SBT, mmHg | 122 ± 22 | 116 ± 23* |  | 124 ± 26 | 116 ± 22* |  | 123 ± 23 | 115 ± 24* |  | 121 ± 23 | 123 ± 25 |
| DBT, mmHg | 58 ± 13 | 60 ± 14 |  | 58 ± 11 | 60 ± 14 |  | 58 ± 13 | 58 ± 13 |  | 57 ± 12 | 61 ± 15* |
| CVP, mmHg | 8 (4 - 11) | 10 (7 - 15)* |  | 6 (4 - 11) | 11 (6 - 16)* |  | 7 (4 - 12) | 10 (6 - 13) |  | 7 (4 - 11) | 10 (7 - 15)* |
| Heart rate, bpm | 82 ± 20 | 91 ± 24* |  | 80 ± 19 | 88 ± 24* |  | 82 ± 21 | 90 ± 23* |  | 86 ± 21 | 81 ± 23* |
| Oxygen saturation, % | 98 (96 - 99) | 98 (95 - 99) |  | 98 (96 - 99) | 97 (95 - 99) |  | 98 (96 - 99) | 97 (96 - 99) |  | 98 (96 - 99) | 97 (96 - 99) |
| Lactate, mmol/L | 1.3 (1 - 1.8) | 1.7 (1.2 - 2.5)* |  | 1.3 (1 - 1.8) | 1.7 (1.2 - 2.6)* |  | 1.3 (1.0 - 1.8) | 1.7 (1.2 - 2.6)* |  | 1.4 (1.0 – 2.0) | 1.4 (1.0 - 2.3) |
| Mechanical ventilation, n (%) | 85 (38) | 28 (42) |  | 53 (36) | 55 (46) |  | 89 (35) | 52 (46)* |  | 89 (37) | 40 (39) |
| PEEP, cmH2O | 8 (8 - 10) | 10 (8 - 10) |  | 8 (7 - 10) | 10 (7 - 10)* |  | 8 (8 - 10) | 10 (7 - 10) |  | 8 (7 - 10) | 8 (7 - 10) |
| Inspiratory peak pressure, cmH2O | 20 (18 - 23) | 22 (18 - 25) |  | 20 (17 - 22) | 21 (19 - 25) |  | 20 (18 - 23) | 21 (17 - 25) |  | 20 (18 - 23) | 21 (17 - 25) |
| pO2/FiO2, kPa | 43 (31 - 54) | 37 (26 - 53)* |  | 43 (33 - 54) | 34 (24 - 47)* |  | 42 (32 - 54) | 34 (24 - 47)* |  | 42 (31 - 55) | 38 (27 - 50)* |
| pO2, kPa | 12.2 (10.6 - 14.9) | 12.9 (9.9 - 14.8) |  | 12.2 (10.8 - 14.7) | 11.7 (9.9 - 14.6) |  | 12.3 (10.8 - 14.9) | 12.3 (9.9 - 14.5) |  | 12.2 (10.7 - 14.7) | 12.1 (9.7 - 14.6) |
| FiO2 | 0.3 (0.25 - 0.37) | 0.35 (0.25 - 0.5)* |  | 0.3 (0.25 - 0.35) | 0.35 (0.28 - 0.5)* |  | 0.3 (0.25 - 0.38) | 0.35 (0.28 - 0.5)* |  | 0.3 (0.25 - 0.38) | 0.3 (0.25 - 0.48)* |
| NA, ug/kg/min | 0 (0 - 0.14) | 0.1 (0 - 0.27)* |  | 0 (0 - 0.13) | 0.05 (0 - 0.24)* |  | 0 (0 - 0.13) | 0.07 (0 - 0.2)* |  | 0.03 (0 - 0.15) | 0 (0 - 0.12) |
| TR Vmax, m/s | 2.5 (2.2–2.8) | 2.7 (2.3–3.0)* |  | 2.4 (1.9 – 3.1) | 2.8 (2.3 – 3.7)* |  | 2.4 (1.9 – 3.1) | 2.8 (2.4–3.7)* |  | 2.5 (1.9 - 3.1) | 2.9 (2.4–3.7)* |
| e´mean, cm/s | 8 (6 - 11) | 6 (4.5 - 8)* |  | 8 (6 - 11) | 6.5 (5 - 8.6)* |  | 8 (6 - 11.5) | 6 (4.5 - 8)* |  | 8.5 (6.5 - 11.5) | 6 (4.6 - 7.5)* |
| E/e | 7.5 (6 - 10.4) | 9.1 (7 - 15.3)* |  | 7.4 (5.8 - 10.4) | 8.6 (6.8 - 12.6)* |  | 7.5 (6.1 - 10.4) | 8.5 (6.4 - 14.6)* |  | 7.1 (5.6 - 9.2) | 10.8 (7.4 - 15.2)* |
| LAVI, cm3 | 26 (19 - 34) | 34 (22 - 47)* |  | 26 (20 - 34) | 29 (20 - 42) |  | 26 (19 - 33) | 34 (22 - 49)* |  | 26 (19 - 33) | 34 (24 - 47)* |
| CI, l/m2 | 3.3 (2.8 - 4.2) | 2.5 (2 - 3.3)* |  | 3.4 (2.9 - 4.2) | 2.6 (2.1 - 3.3)* |  | 3.4 (2.8 - 4.4) | 2.7 (2.0 - 3.3)* |  | 3.4 (2.8 - 4.4) | 2.7 (2.1 - 3.4)* |
| SVI, ml/m2 | 45 (36 - 53) | 28 (23 - 36)* |  | 46 (39 - 54) | 32 (25 - 41)* |  | 45 (37 - 54) | 31 (25 - 39)* |  | 43 (35 - 52) | 36 (28 - 48)* |
| VTI, cm | 21.5 (18.1 - 26.1) | 14.7 (11.9 - 18.4)* |  | 23 (19.8 - 26.7) | 17 (13 - 20)* |  | 21.7 (18.9 - 26.3) | 15.8 (12.6 - 19.4)* |  | 21.2 (18.1 - 26) | 18.3 (14.4 - 23)* |
| TAPSE, cm | 2.2 (1.9 - 2.6) | 1.7 (1.2 - 2.1)* |  | 2.3 (2 - 2.7) | 1.8 (1.4 - 2.1)* |  | 2.2 (1.9 - 2.6) | 1.7 (1.3 - 2)* |  | 2.2 (1.9 - 2.6) | 1.8 (1.3 - 2.1)* |
| FRAC, % | 44 (38 - 50) | 37 (24 - 47)* |  | 44 (39 - 50) | 39 (31 - 47)* |  | 44 (38 - 50) | 37 (26 - 46)* |  | 44 (39 - 51) | 39 (26 - 43)* |

**Supplemental Table 1. Clinical and echocardiographic characteristics according to dichotomised left ventricular systolic function parameters**

MAP, mean arterial pressure; SBT, systolic blood pressure; DBT, diastolic blood pressure; CVP, central venous pressure; PEEP, positive end-expiratory pressure; NA, norepinephrine infusion rate; TR Vmax, maximal tricuspid regurgitation velocity; e′mean, mean early diastolic mitral annular velocity; E/e′, ratio of transmitral E- wave to mitral annular e′ velocity; LAVI, left atrial volume index; CI, cardiac index; SVI, stroke volume index; VTI, velocity–time integral; TAPSE, tricuspid annular plane systolic excursion; FRAC, right ventricular fractional area change.

**Supplemental Table 2. Associations between echocardiographic parameters and 90-day mortality according to clinical subgroup.**

| **Sepsis,**  **n=118** |  |  |  |  |  |  |  |  |
| --- | --- | --- | --- | --- | --- | --- | --- | --- |
|  |  | Unadjusted | | |  | Adjusted* | | |
| Category | Echocardiographic parameter | OR | 95% CI for OR | p-value |  | OR | 95% CI for OR | p-value |
| Continuous | Simpson biplane, per 10% decrease | 1.00 | 0.73 - 1.37 | 0.989 |  | 0.89 | 0.61 - 1.31 | 0.558 |
|  | GLS, per % increase | 1.09 | 0.98 - 1.22 | 0.103 |  | 1.04 | 0.91 - 1.18 | 0.582 |
|  | MAPSE, per mm decrease | 1.11 | 0.99 - 1.25 | 0.093 |  | 1.08 | 0.93 - 1.25 | 0.326 |
|  | S', per cm/s decrease | 1.02 | 0.89 - 1.16 | 0.818 |  | 0.93 | 0.79 - 1.12 | 0.490 |
| Dichotomized | Simpson biplane <50% | 0.90 | 0.32 - 2.51 | 0.836 |  | 0.61 | 0.18 - 2.11 | 0.434 |
|  | GLS > -16% | 2.50 | 0.94 - 6.65 | 0.066 |  | 1.68 | 0.54 - 5.26 | 0.374 |
|  | MAPSE ≤10mm | 2.33 | 1.08 - 5.03 | 0.031 |  | 1.90 | 0.73 - 4.92 | 0.188 |
|  | S' ≤7.5cm/s | 1.12 | 0.50 - 2.54 | 0.781 |  | 0.62 | 0.22 - 1.74 | 0.363 |

| **Respiratory failure n=184** |  |  |  |  |  |  |  |  |
| --- | --- | --- | --- | --- | --- | --- | --- | --- |
|  |  | Unadjusted | | |  | Adjusted* | | |
| Category | Echocardiographic parameter | OR | 95% CI for OR | p-value |  | OR | 95% CI for OR | p-value |
| Continuous | Simpson biplane, per 10% decrease | 1.31 | 1.03 - 1.68 | 0.03 |  | 1.09 | 0.8 - 1.49 | 0.574 |
|  | GLS, per % increase | 1.16 | 1.07 - 1.26 | <.001 |  | 1.07 | 0.96 - 1.19 | 0.218 |
|  | MAPSE, per mm decrease | 1.24 | 1.11 - 1.38 | <.001 |  | 1.12 | 0.98 - 1.28 | 0.085 |
|  | S', per cm/s decrease | 1.14 | 1.01 - 1.29 | 0.029 |  | 1.09 | 0.92 - 1.29 | 0.306 |
| Dichotomized | Simpson biplane <50% | 1.98 | 0.92 - 4.27 | 0.082 |  | 1.12 | 0.43 - 2.94 | 0.816 |
|  | GLS > -16% | 3.87 | 1.71 - 8.76 | 0.001 |  | 1.70 | 0.60 - 4.88 | 0.321 |
|  | MAPSE ≤10mm | 3.50 | 1.83 - 6.69 | <.001 |  | 1.58 | 0.68 - 3.64 | 0.285 |
|  | S' ≤7.5cm/s | 1.95 | 1.00 - 3.81 | 0.050 |  | 1.21 | 0.48 - 3.10 | 0.687 |

| **Cardiac disease, n=58** |  |  |  |  |  |  |  |  |
| --- | --- | --- | --- | --- | --- | --- | --- | --- |
|  |  | Unadjusted | | |  | Adjusted* | | |
| Category | Echocardiographic parameter | OR | 95% CI for OR | p-value |  | OR | 95% CI for OR | p-value |
| Continuous | Simpson biplane, per 10% decrease | 1.03 | 0.71 - 1.51 | 0.873 |  | 0.79 | 0.48 - 1.31 | 0.367 |
|  | GLS, per % increase | 1.12 | 0.97 - 1.30 | 0.109 |  | 1.03 | 0.86 - 1.24 | 0.719 |
|  | MAPSE, per mm decrease | 1.19 | 0.99 - 1.42 | 0.047 |  | 1.11 | 0.89 - 1.39 | 0.331 |
|  | S', per cm/s decrease | 1.04 | 0.84 - 1.27 | 0.741 |  | 0.91 | 0.70 - 1.18 | 0.478 |
| Dichotomized | Simpson biplane <50% | 0.87 | 0.27 - 2.84 | 0.813 |  | 0.30 | 0.06 - 1.47 | 0.138 |
|  | GLS > -16% | 1.60 | 0.35 - 7.23 | 0.541 |  | 0.49 | 0.07 - 3.62 | 0.485 |
|  | MAPSE ≤10mm | 2.53 | 0.81 - 7.89 | 0.111 |  | 1.45 | 0.36 - 5.88 | 0.605 |
|  | S' ≤7.5cm/s | 1.20 | 0.37 - 3.92 | 0.763 |  | 0.39 | 0.06 - 1.95 | 0.225 |

| **Other,  n=107** |  |  |  |  |  |  |  |  |
| --- | --- | --- | --- | --- | --- | --- | --- | --- |
|  |  | Unadjusted | | |  | Adjusted* | | |
| Category | Echocardiographic parameter | OR | 95% CI for OR | p-value |  | OR | 95% CI for OR | p-value |
| Continuous | Simpson biplane, per 10% decrease | 0.53 | 0.20 - 1.43 | 0.211 |  | 0.6 | 0.16 - 2.3 | 0.459 |
|  | GLS, per % increase | 1.02 | 0.85 - 1.23 | 0.828 |  | 0.95 | 0.75 - 1.22 | 0.703 |
|  | MAPSE, per mm decrease | 1.68 | 1.25 - 2.26 | <.001 |  | 1.41 | 0.87 - 2.27 | 0.161 |
|  | S', per cm/s decrease | 1.51 | 1.10 - 2.10 | 0.012 |  | 1.83 | 1.03 - 3.27 | 0.041 |
| Dichotomized | Simpson biplane <50% | - | - | - |  | - | - | - |
|  | GLS > -16% | 1.73 | 0.31 - 9.85 | 0.535 |  | - | - | - |
|  | MAPSE ≤10mm | 14.7 | 2.78 - 77.5 | 0.002 |  | 5.77 | 0.45 - 74.8 | 0.180 |
|  | S' ≤7.5cm/s | 4.51 | 1.12 - 18.2 | 0.034 |  | 8.88 | 1.0 - 78.9 | 0.050 |

* adjusted for age, SAPS 3 and Cardiac index**Supplemental Analysis 1**
Nested model analyses using the −2 log-likelihood (−2LL) in patients with complete datasets for GLS, MAPSE, and cardiac index (n = 234).

Model 1: Baseline model (SAPS 3, age, and cardiac index)

- −2LL: 183.84

Model 2A: Baseline + GLS

- −2LL: 180.56
- Δ−2LL = 3.28, p = 0.070

Model 2B: Baseline + MAPSE

- −2LL: 178.58
- Δ−2LL = 5.26, p = 0.022

Model 3: Baseline + MAPSE + GLS

- −2LL: 178.28
- Addition of GLS to the MAPSE model: Δ−2LL = 0.28, p = 0.58
- Addition of MAPSE to the GLS model: Δ−2LL = 2.28, p = 0.13
